# A Method to Directly Identify *Cronobacter sakazakii* in Liquid Medium by MALDI-TOF MS

**DOI:** 10.3390/foods12101981

**Published:** 2023-05-12

**Authors:** Danliangmin Song, Qunchao Su, Ai Jia, Shiqian Fu, Xiaoming Ma, Tiantian Li, Chaoxin Man, Xinyan Yang, Yujun Jiang

**Affiliations:** 1 Department of Food Science, Northeast Agricultural University, Harbin 150038, China; 2 Key Laboratory of Animal Protein Food Deep Processing Technology of Zhejiang Province, College of Food and Pharmaceutical Sciences, Ningbo University, Ningbo 315800, China; 3 Key Laboratory of Dairy Science, Ministry of Education, Harbin 150030, China

**Keywords:** *Cronobacter sakazakii*, MALDI-TOF MS, liquid spotting, response surface methodology, direct identification

## Abstract

Matrix-assisted laser desorption ionization time-of-flight mass spectrometry has been widely used as an emerging technology for the rapid identification of microorganisms. *Cronobacter sakazakii (C. sakazakii)* is a food-borne pathogen of particular importance to the powdered infant formula (PIF) processing environment due to its high lethality in infants. However, the traditional solid spotting detection method of pretreating samples for MALDI-TOF MS leads only to qualitative detection of *C. sakazakii*. We developed a new, low-cost, robust liquid spotting pretreatment method and used a response surface methodology to optimize its parameters. The applicability, accuracy, and quantitative potential were measured for different types of samples. The optimal parameters of this method were as follows: a volume of 70% formic acid of 25 μL, treatment with ultrasound at 350 W for 3 min, and a volume of acetonitrile added of 75 μL. These conditions led to the highest identification score for *C. sakazakii* (1926.42 ± 48.497). This method was found to detect bacteria accurately and reproducibly. When 70 strains of *C. sakazakii* isolates were analyzed with this method, the identification accuracy was 100%. The detection limit of *C. sakazakii* in environmental and PIF samples was 4.1 × 10^1^ cfu/mL and 2.72 × 10^3^ cfu/mL, respectively.

## 1. Introduction

Food-borne pathogens are of intense interest, as they are often strongly pathogenic and can persist in a variety of environments [1,2,3]. Among these pathogens, *C. sakazakii* is of particular importance to the infant formula industry due to its high lethality in infants and because infant formula manufacturing plants are susceptible to contamination [4,5]. Accordingly, industries dealing with the production and processing of powdered infant formula (PIF) tend to be highly regulated throughout the world.

Effective control of *C. sakazakii* can be established by ensuring rapid throughput of susceptible materials. In addition, continuous monitoring of the processing environment can provide regulators with an understanding of the level of risk present in the final product. However, incidents of *C. sakazakii* contamination of PIF continue to emerge globally without interruption [6,7,8,9]. There is an urgent need to develop a rapid and reliable identification method to monitor *C. sakazakii* in PIF processing environments and in final products.

Traditional pathogen detection methods, such as biochemical [10], colorimetric [11], and enzyme-linked immunosorbent assays [12], are limited due to their cumbersome operations and requirements for highly trained technicians [13]. Other problems, including challenges with distinguishing similar species and genera and the identification of microorganisms that are difficult to cultivate, further make these methods less than optimal [14,15].

Matrix-assisted laser desorption ionization time-of-flight mass spectrometry (MALDI-TOF MS) is an emerging technique in the field of microbial detection [16]. Due to its cost- and time-effectiveness and its amenability to high-throughput applications, MALDI-TOF MS has been widely used in the identification of pathogenic microorganisms and targeted antibiotic therapy [17,18]. The identification of microorganisms by MALDI-TOF MS is typically performed by spotting single colonies on solid medium; this method can lead to errors in identification results [19]. The solid spotting method is also time-consuming as compared with traditional methods of pathogenic bacteria detection, as it can take up to 12 to 18 h. Other disadvantages of this method include: (1) the results can be influenced by the pretreatment conditions, resulting in poor reproducibility; (2) some microbes produce small colonies that are difficult to work with; and (3) the detection results only permit qualitative identification and do not permit quantitative analyses [20].

The methods by which MALDI-TOF MS has been used for the detection and identification of pathogenic bacteria are divided into the establishment of a library of pathogenic bacteria and the development of pretreatment methods for liquid spotting [21,22,23]. Although the application of MALDI-TOF MS, in combination with appropriate pretreatment steps, to the identification of microorganisms growing in liquid culture media has been pursued in a few studies, the solid spotting technique is still used almost exclusively. Current liquid techniques require cell densities of more than 10^5^ cfu/mL. In addition, these methods cannot properly be called liquid direct spotting methods because they involve pre-treating bacteria that have been cultured in liquid, but traditional methods are still used to pick out a bacterial pellet for spotting [24,25,26].

In order to advance the use of MALDI-TOF MS as a microbial identification tool, we developed and optimized a new pretreatment method for the direct identification of bacteria from liquid culture media. The research idea of this study is based on the defects of traditional MALDI-TOF MS microbial identification combined with the research progress of MADLI-TOF MS on the detection and quantification of hazardous substances and adulteration in multi-class food samples [27,28]. To explore the feasibility of MALDI-TOF MS for the identification and detection of pathogenic bacteria, which is very rare at present. There are also several similarities in MALDI-TOF MS analysis (intensity of peak) between cell numbers (cfu/mL). *C. sakazakii* was used as the representative pathogen, and the method was optimized with a response surface methodology. After optimization, the detection accuracy, repeatability, applicability, and direct detection ability of the established method were evaluated. This study provides a new method for the detection of *C. sakazakii* in samples from PIF production environments and in PIF samples.

## 2. Materials and Methods

### 2.1. Materials

Luria-Bertani (LB), nutrient broth (NB), and tryptic soy broth (TSB) media were purchased from Beijing Soleibo Biotechnology Co., Ltd. (Beijing, China) Acetonitrile (ACN), α-cyano-4-hydroxycinnamic acid (CHCA), and formic acid (FA) were purchased from Intelligene Biosystems (Qingdao Co., Ltd., Qingdao, China). Trifluoroacetic acid and anhydrous ethanol were purchased from Jinzhiyuan Chemical Reagent Co., Ltd. (Tianjing, China). A bacterial genomic DNA rapid extraction kit was purchased from BioTeKe Company (Beijing, China). All other solvents were analytical grade and purchased locally.

### 2.2. Bacterial Strains and Cultivation Conditions

This study involved *C. sakazakii* ATCC 29544 and several other common pathogens relevant to the production and processing of PIF. Standard strains of *Cronobacter* spp. (including *C. universalis*, *C. turicensis*, *C. muytjensii*, *C. dublinensis,* and *C. malonaticus*) were used to compare the performance of analytical methods involving solid spotting and liquid spotting. The 66 *C. sakazakii* strains used in this study were all isolated from various environments within PIF production facilities, specifically fluidized beds, fixed beds, and U-shaped valves, as well as spray-dried powder samples. These PIF-related cultures were used to evaluate the accuracy of direct identification from liquid culture media. All strains were cultured in LB liquid media overnight at 37 °C with shaking at 150 rpm. Cells in these cultures were isolated by centrifugation at 12,000× *g* for 2 min at 4 °C, and the pellets were resuspended in sterilized PBS (0.1 M, pH 7.4). The number of bacterial cells was determined by the TSB agar plating method.

### 2.3. Direct Identification of C. sakazakii from Liquid Culture Media Pretreatment Method Process by MALDI-TOF MS

**Figure 1:**
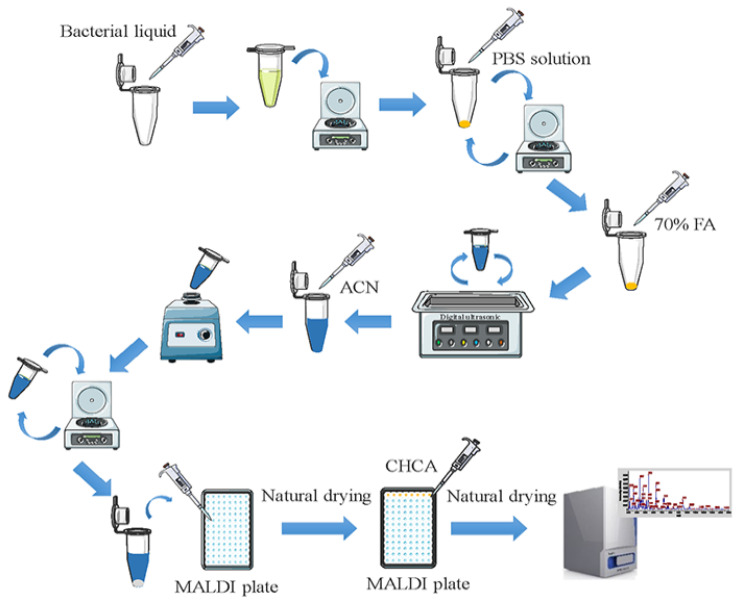
Overall flow of the MALDI-TOF MS liquid pretreatment spotting method.

Pretreatment of samples was performed by transferring 500 μL of a suspension containing bacteria at a density of 10^8^ cfu/mL into a 1.5 mL sterile centrifuge tube, which was centrifuged at 12,000× *g* for 2 min. The supernatant was removed, and the pellet was washed twice with 500 μL of sterile PBS. FA was added, and the mixture was subjected to ultrasonic disruption. ACN was then added, and the mixture was mixed by vortexing for 2 min and then centrifuged at 12,000× *g* for 2 min. A sample (1.5 μL) of the supernatant was applied to the target plate and allowed to incubate for 10 min to air dry. Next, an aliquot (1.5 μL) of a MALDI matrix solution containing CHCA was added, and the mixture was air dried prior to mass spectrometric analysis. The overall process is shown schematically in Figure 1.

### 2.4. Single-Factor Test

The parameters that were analyzed as single factors were the volume of 70% FA added (10, 20, 30, 40, and 50 μL), the ultrasound power (300, 350, 400, 450, and 500 W), the time of ultrasound exposure (1, 2, 3, 4, and 5 min), and the volume of ACN added (20, 40, 60, 80, and 100 μL). The MALDI-TOF MS spectral data acquisition was performed under the following conditions: laser energy, 5 μJ; laser frequency, 5000 Hz; detector voltage, −0.56 kV; and focus mass, 10,000 u. The spectral data were imported into the QuanID DB database for comparison, and the identification of microorganisms was accomplished by analyzing the mass-to-charge ratios of characteristic peaks. The scores that represented the accuracy of the identification were used to determine the optimal parameters for each of the single factors. Each experiment was repeated three times.

### 2.5. Response Surface Methodology

According to the optimal parameters determined in the single factor experiments and based on the Box-Behnken experimental design principle, a response surface analysis method with four factors and three levels was designed (Table 1). Using the MALDI-TOF MS microbial identification score as the evaluation index, the parameters used for sample pretreatment of liquid cultures prior to MALDI-TOF MS were tested systematically. Each experiment was repeated three times. Values generated in two conditions were compared using one-way ANOVA with SPSS 17.0, and the least significant difference test was used to compare multiple results.

### 2.6. Evaluation of the Accuracy of Identification of the MALDI-TOF MS Liquid Spotting Pretreatment Method

The effects of different types of liquid growth media (LB, NB, and TSB) on the identification method were analyzed using identical culture conditions. In this series of experiments, 66 strains of *C. sakazakii* that were isolated from different sources and four reference strains of *C. sakazakii* were subjected to the identification method utilizing the optimized liquid spotting pretreatment method. The QuanID DB microbial database was used as a reference for this evaluation.

### 2.7. Comparison of Solid and Liquid Spotting Methods

Strains of *Cronobacter* spp. were streaked onto solid agar and incubated for 12 h. The solid spotting method was performed by using a 1 μL inoculation loop to isolate a single colony from this plate. This colony was transferred to a target plate, and 2 μL of 70% FA was added dropwise. After the spot was air-dried, MALDI matrix solution (1.5 μL) was added dropwise, and mass spectrometric identification was performed after this sample was dry. The liquid spotting method was performed using each optimized parameter as described above. The two methods were compared using cluster analyses of the spectral data as performed using Origin 2018 software (version number: OriginPro 2018C).

### 2.8. Direct Detection and Analysis of the Linear Relationship between the Cell Number of C. sakazakii in Environmental Samples

The detection of *C. sakazakii* simulated in environmental samples is performed by adding *C. sakazakii* cells to sterile PBS by gradient dilution as follows: Cells obtained during the collection of environmental pathogens or the reference strain *C. sakazakii* ATCC 29544 were subjected to serial 10-fold dilutions in sterile PBS to obtain 1 mL samples with cell numbers ranging from 10^8^ to 10^0^ cfu/mL. Identification was carried out by MALDI-TOF MS using the optimized liquid pretreatment method, and the detection limit was determined. Under the culture conditions of 37 °C and 150 rpm, using TSB, LB, and NB liquid medium to optimize the pre-enrichment medium, medium addition amount (400, 600, 800, and 1000 μL), and pre-enrichment time (0, 2, 4, and 6 h), and finally determine the optimal conditions for *C. sakazakii* ATCC 29544 in environmental collection samples. According to the linear regression equation with the number of *C. sakazakii* cells as the abscissa and the peak intensity in the obtained identification spectrum as the ordinate, each experiment was repeated three times. The relative standard deviation (RSD) was calculated to evaluate the repeatability and stability of the method.

### 2.9. Direct Detection and Analysis of the Linear Relationship between the Cell Number of C. sakazakii in PIF Samples

The feasibility of simulating the MALDI-TOF MS pretreatment method for the number of *C. sakazakii* cells in collected PIF samples is as follows: Samples of PIF (20 mg/mL) verified to be free of *C. sakazakii* contamination were spiked with a target strain of bacteria. The samples were serially diluted 10-fold in sterile PBS to generate samples containing various cell densities, from 10^8^ to 10^0^ cfu/mL. The cells were collected by centrifugation for 2 min at 12,000× *g*, and the pellets were added to 1 mL samples of commercial PIF. After a pre-enrichment incubation for 0, 2, 4, or 6 h at 37 °C and shaking at 150 rpm, these samples were mixed by vortexing. Portions (500 μL) of the resulting samples were centrifuged at 5000× *g* for 5 min, and the supernatants were removed. The pellets were washed only several times with sterile PBS to completely eliminate the lipid layer. The other components in the PIF sample are not further isolated and purified, minimizing the loss of *C. sakazakii* cell numbers. MALDI-TOF MS identification was then performed using the optimized liquid pretreatment method. The peak intensities in the resulting spectra were plotted as a function of the number of *C. sakazakii* cells, and the linear regression equation defining the relationship was obtained. Each experiment was repeated three times. The RSD was calculated to evaluate the repeatability and stability of the method.

## 3. Results and Discussion

### 3.1. Effects of Single Factors on the Identification Score of C. sakazakii from Liquid Culture Media by MALDI-TOF MS

**Figure 2:**
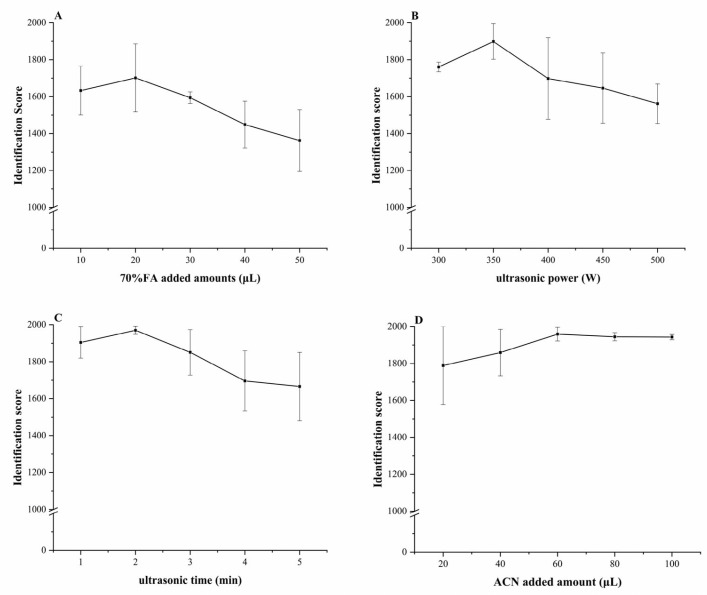
Effects of single factors on the identification score of *C. sakazakii* from liquid culture media by MALDI-TOF MS. (**A**) Effect of 70% FA on the identification score of *C. sakazakii* by MALDI-TOF MS. (**B**) Effect of ultrasonic power on the identification score of MALDI-TOF MS. (**C**) Effect of ultrasonic time on the identification score of *C. sakazakii* by MALDI-TOF MS. (**D**) Effect of ACN on the identification score of *C. sakazakii* by MALDI-TOF MS.

The effects of several pretreatment factors were tested individually. Specifically, these factors were the amount of 70% FA added, the power of the ultrasound used to disrupt the cells, the time of ultrasonic treatment, and the amount of ACN added. When the MALDI-TOF MS technique was used to process the cultures, the accuracy of the identification was assigned a score. The relationships between this score and each factor are shown in Figure 2.

As shown in Figure 2A, the MALDI-TOF MS identification score of *C. sakazakii* in liquid culture was optimal when the volume of 70% FA added was 20 μL. When the amount of 70% FA exceeded 20 μL, the identification score was significantly lower than the score obtained following the addition of 20 μL of 70% FA (*p* < 0.05). This negative impact of excess FA addition may be due to the degradation of ribosomal proteins released by *C. sakazakii* upon lysis of the cell membrane, resulting in differences in the experimental and reference spectra. In support of this mechanism, higher volumes of 70% FA led to fewer ribosomal protein matches, resulting in a lower identification score. Therefore, 20 μL was selected as the optimal amount of 70% FA to be added.

The relationship of identification score with ultrasonic power showed an upward trend between 300 and 350 W, which may be due to increased cavitation and enhanced cellular lysis. An increased permeability of *C. sakazakii* cells would be expected to increase the release of ribosomal proteins. When ultrasound was applied at 350 W, the outer membrane of the cell was likely completely broken, resulting in complete dissolution of the ribosomal proteins and maximization of the MALDI-TOF MS identification score. Higher ultrasonic power may have changed or destroyed ribosomal proteins, affecting the positions of their correlated peaks in the spectrum and thereby reducing the identification score [29]. Therefore, 350 W was selected as the optimal pretreatment ultrasonic power.

The effect of the time of ultrasound treatment on the MALDI-TOF MS identification of *C. sakazakii* in liquid culture was also investigated (Figure 2C). The identification scores first increased until the treatment time reached 2 min, and then the score decreased with longer processing times until it ultimately reached a stable value. While longer ultrasound treatments would be expected to result in a more efficient release of ribosomal proteins, the thermal effect caused by continuous sonication may lead to the degradation of certain large-molecular-weight proteins, thereby affecting the accuracy of identification [30]. Therefore, an ultrasonic treatment time of 2 min was determined to be optimal.

As shown in Figure 2D, the influence of the volume of ACN addition on the identification score showed an upward trend in the range of 20 to 60 μL. At 60 μL ACN, the identification score reached a plateau that continued over a range of 60 to 100 μL ACN. These results suggest that 60 μL of ACN leads to complete disruption of the membrane and release of the ribosomal proteins of *C. sakazakii*, as adding additional ACN did not significantly improve the identification score. In consideration of reagent cost minimization, the optimal volume of ACN was 60 μL.

### 3.2. Optimization of Direct Identification of C. sakazakii from Liquid Culture Media by MALDI-TOF MS by RSM

Response surface methodology was utilized to collectively optimize the pretreatment parameters. Design-Expert 8.0.6.1 software was used to perform regression analysis on the test data. The Box-Behnken four-factor, three-level test had a total of 29 experimental points. The first 24 were factorial points; here, the independent variables were the three-dimensional vertices composed of volume of 70% FA, ultrasonic power, ultrasonic time, and volume of ACN. The last five zero points, which represent the center of the region, were used to evaluate the experimental error. The MALDI-TOF MS liquid culture medium microorganism direct identification method Box-Behnken test scheme and results are shown in Table 2.

### 3.3. Statistical Analysis and Modeling of Direct Identification of C. sakazakii from Liquid Culture Media by MALDI-TOF MS

Design-Expert 8.0.6.1 software was used to perform multiple regression fitting on the data displayed in Table 2 to obtain a quadratic multinomial regression model. In this model, the MALDI-TOF MS identification score (y) was found to depend on the volume of 70% FA added in μL (A), ultrasonic power in W (B), ultrasonic time in min (C), and volume of CAN added in μL (D) according to the equation: y = 137.51 − 24.31A + 6.53B − 487.22C + 38.46D + 0.13AB + 7.28AC + 0.39AD + 1.03BC − 0.08BD + 6.64CD − 1.49A^2^ − 0.009B^2^ − 99.80C^2^ − 0.23D^2^. The correlation of this model was extremely significant (*p* < 0.01), and the lack-of-fit term was not significant (*p* > 0.05), indicating that the equation fits well with experimental results. We conclude that the MALDI-TOF MS technique can be used to directly identify microorganisms grown in liquid culture under a variety of pretreatment conditions.

The results of the test of significance for the regression equation coefficients are shown in Table 3. The order of influence of each factor on the identification score of *C. sakazakii* was: volume of ACN added (D) > ultrasonic time (C) > volume of 70% FA added (A) > ultrasonic power (B). Among these factors, the effect of A^2^ was extremely significant (*p* < 0.01), and the effects of C^2^ and D^2^ were significant (*p* < 0.05).

### 3.4. Analysis of Response Surface and Two-Dimensional Contour Plots

**Figure 3:**
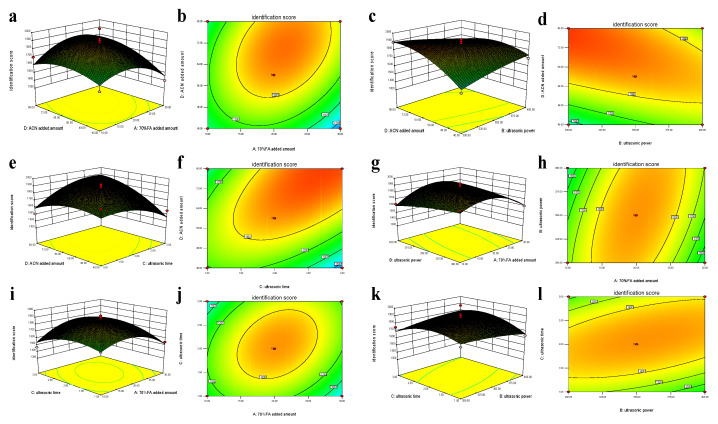
Influence of different factors on the identification score of *C. sakazakii* by the MALDI-TOF MS liquid spotting pretreatment method. (**a**,**b**) The effects of the volume of 70% FA added and the volume of ACN added on the identification scores. (**c**,**d**) The effects of the volume of ACN added and ultrasonic power on the identification scores; (**e**,**f**) The effects of the ultrasonic time and volume of ACN added on the identification score; (**g**,**h**) The effects of the volume of 70% FA added and ultrasonic power on the identification scores; (**i**,**j**) The effects of the ultrasonic treatment time and volume of 70% FA added; (**k**,**l**) The effects of the ultrasonic time and ultrasonic power on the identification scores.

Two-dimensional contour plots were developed as graphical representations of the regression model (Figure 3). The shapes of such plots can be used as an indication of the significance of the interactions between two tested variables, where circular contour plots indicate that the interactions are not significant and elliptical or saddle-shaped contour plots suggest that the interactions are significant.

Figure 3a,b compare the effects of the volume of 70% FA added and the volume of ACN added on the identification scores. In these plots, when the amount of FA was constant, the MALDI-TOF MS identification score was affected by the volume of ACN added; here, the identification score increased until it reached a maximum and then decreased. The contour arrangement of the relationship between these two factors was relatively loose, and the elliptic curvature of the plot was small, indicating that the interaction between the two factors was not significant.

The effects of the volume of ACN added and ultrasonic power on the identification score were also tested (Figure 3c,d). Here, the MALDI-TOF MS identification score increased with the addition of ACN when the ultrasonic power was kept constant. The contour lines of the ACN addition axis are more dense than those on the ultrasonic power axis, indicating that ACN addition had a greater impact on the MALDI-TOF MS identification score and that the impact of ultrasonic power on the identification score was small [31]. When tested as single factors, the influences of ultrasonic time and volume of ACN added on the identification score were found to be the same, as both increased up to a maximum point and then decreased (Figure 3e,f).

As is evident from Figure 3g,h, the contour line of the axis relating to the volume of 70% FA added is significantly denser than the contour line of the ultrasonic power axis, indicating that the effect of FA addition on the identification score is greater than that of the ultrasonic power. The effect of ultrasonic treatment time on the MALDI-TOF MS identification score tends to increase first and then decrease, and vice versa (Figure 3i,j). These data also demonstrate that the interaction between these two factors was not significant (*p* > 0.05). Similarly, when the ultrasonic time was held constant and the ultrasonic power was increased, the slope of the graph of the relationship to the identification score was negative (Figure 3k,l), indicating that the score increased. This lower identification score may be due to disruption of ribosomal proteins due to the thermal effect of excessive sonication time or power [30].

### 3.5. Optimization of Pretreatment Parameters and Validation of the Optimized Conditions

When we analyzed our model with Design-Expert 8.0.6.1 software, the optimal pretreatment conditions for the liquid spotting MALDI-TOF MS method were found to be: 25.62 μL of 70% FA added, ultrasonic power of 359.15 W, time of ultrasonic of 3 min, and 75 μL of ACN added. These conditions led to an identification score of 1926.42 when applied to a liquid culture of *C. sakazakii*. To test the reliability of the results from the response surface method, the optimal pretreatment conditions were applied to identification tests using other strains grown in liquid culture. In consideration of the practical limitations of the procedure, the pretreatment parameters were adjusted to 25 μL of 70% FA added, 350 W of ultrasonic power, 3 min of ultrasonic treatment time, and 75 μL of ACN added.

Three parallel tests were carried out under the adjusted conditions. The average strain identification score was determined to be 1972.00 ± 23.356 after the liquid sample pretreatment method. This value was consistent with the theoretical value of 1926.42 ± 48.497, for a relative deviation of 2.3%. In other words, our results demonstrated that the response surface method optimization model was well fitted with the experimental observations, indicating that the model is reliable.

### 3.6. Evaluation of the Accuracy of Identification Using the Liquid Spotting Pretreatment Method

The growth and metabolism of microorganisms can be affected by the composition of the growth medium [32]. Therefore, we endeavored to investigate the influence of different medium components on the identification score of the MALDI-TOF MS microbial liquid spotting method. Here, we cultivated *C. sakazakii* ATCC 29544 to a density of 10^8^ cfu/mL in different media mixtures (LB, NB, and TSB). We then used the optimized processing method for MALDI-TOF MS-based identification.

The MALDI-TOF MS identification scores for this comparison are shown in Table 4. There was no significant difference in the identification scores of cultures grown in different media (*p* > 0.05). This result indicates that the composition of the medium used to culture target microorganisms is not an important factor in the identification process.

The optimized MALDI-TOF MS liquid pretreatment spotting method (25 μL of 70% FA, 350 W of ultrasonic power, 3 min of ultrasonic treatment time, and 75 μL of ACN) was used to attempt to identify 66 strains from different PIF-related sources and four reference strains of *C. sakazakii*. Of the 66 community strains, a total of 50 came from northeastern China; these strains include 24 isolated from commercially available PIF, 15 isolated from samples from PIF plants, and five isolated from fluidized powders at PIF plants, as well as 22 other strains isolated from various areas within PIF plants. Each of the 66 strains was identified both with the MALDI-TOF MS liquid spotting method and with a conventional method based on determining the sequence of 16S rDNA. The results obtained by MALDI-TOF MS were consistent with those obtained from 16S rDNA studies for all 66 samples tested (Table 5). Therefore, we conclude that the MALDI-TOF MS liquid pretreatment method that was developed and optimized can be applied to the identification of *C. sakazakii* strains from various sources.

### 3.7. Comparison of Solid Spotting/Liquid Spotting Methods

**Figure 4:**
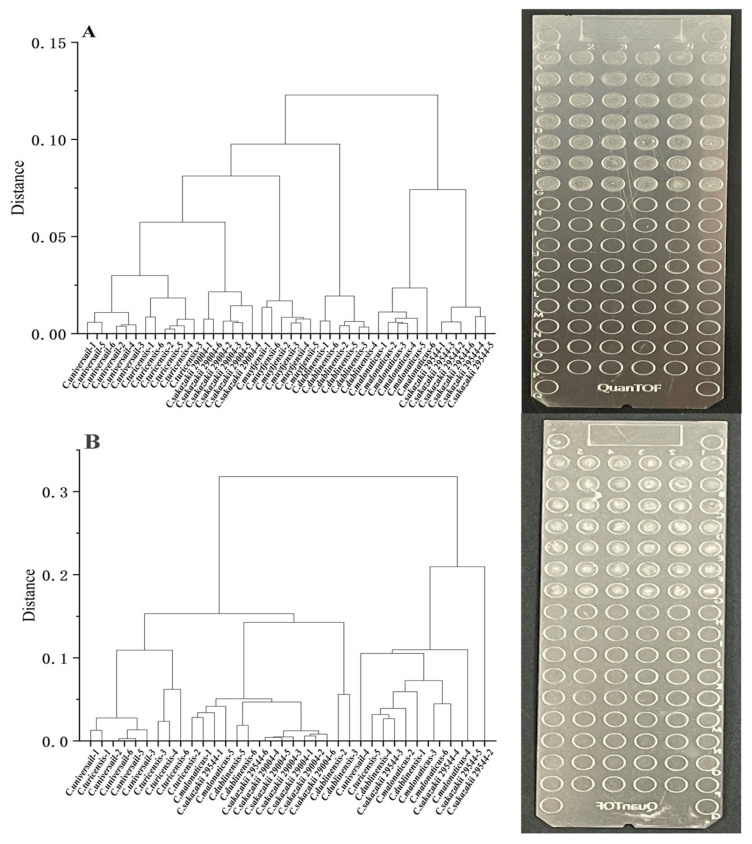
Comparison of MALDI-TOF MS solid/liquid spotting methods. (**A**) Clustering analysis of liquid spotting method data and target morphology. (**B**) Clustering analysis of solid spotting method data and target morphology.

The reproducibility of the liquid spotting pretreatment method was evaluated with a clustering analysis. Clustering is the process of categorizing data into different classes or clusters, permitting a comparison of the similarity of individual data points and thus how well a method of microbial identification is able to distinguish similar subjects [33]. This strategy was used to investigate the reproducibility of the liquid spotting pretreatment method and the ability of the method to differentiate different organisms. Figure 4 shows the results of cluster analyses that compared the MALDI-TOF MS spectral data generated from *Cronobacter* spp. at different species levels with data generated from different strains at the same species level.

As shown in Figure 4A, after pretreatment using the liquid spotting technique, the different species levels that characterize *Cronobacter* spp. can be well distinguished, and the results have good reproducibility. Compared with the cluster analysis data generated from liquid spotting (Figure 4A), lower reproducibility was observed in a cluster analysis of MALDI-TOF MS data generated using the traditional solid spotting method (Figure 4B). In addition, it was impossible to distinguish different levels and different strains of microorganisms using the solid spotting method. This result is somewhat different from the results obtained by Wang et al. [34]. They treated single colonies of *Cronobacter* cultured on TSA medium using the ethanol/formic acid method. Although different levels of *Cronobacter* can be distinguished, this is based on microbial colonies. The data were not uniform, and the thickness of the target plate site was found to be variable, suggesting that it is strongly dependent on the spotting method. These inconsistent results may be due in part to the fact that some microbial colonies formed are small and therefore difficult to spot and detect in the operation of the identification method.

### 3.8. Identification of the Analysis Relationship between the Cell Number of C. sakazakii and Peak Intensities Using Environmental and PIF Samples

**Figure 5:**
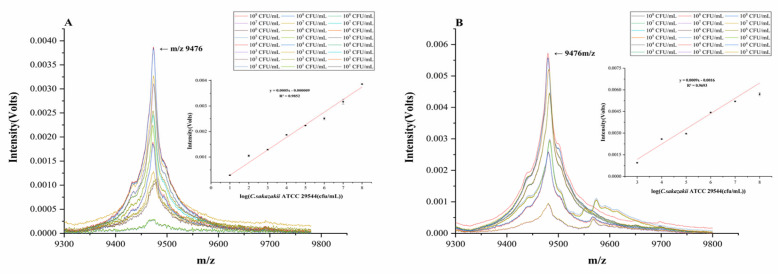
MALDI spectra of the relative intensity of *C. sakazakii* at different numbers of bacterial cells in environmental and PIF samples. MALDI spectra of the relative intensity of *C. sakazakii* at different numbers of bacterial cells in environmental (**A**) and PIF samples (**B**).

The MALDI-TOF MS liquid pretreatment and sample loading method was used to detect *C. sakazakii* contamination in samples from the production environment and in samples of PIF spiked with the microorganism. Using pre-treatment methods without pre-enrichment, the detection limit was found to be 1.18 × 10^6^ cfu/mL. We then determined whether a pre-enrichment method would improve detection. After pre-enrichment culturing of *C. sakazakii* in different liquid media, MALDI-TOF MS detection was carried out by the liquid spotting pretreatment method. Among the types of media tested, we focused on the use of LB liquid medium (600 μL) and performed the detection procedure after pre-enrichment for 6 h; in this case, the detection limit reached 4.1 × 10^1^ cfu/mL. When TSB and NB liquid media were used for pre-enrichment, the detection limit reached 1.42 × 10^2^ cfu/mL after 6 h of pre-enrichment. By analyzing the MALDI-TOF MS spectra generated from samples with different initial numbers of *C. sakazakii* cells (10^1^–10^8^ cfu/mL) after pre-enrichment (Figure 5A), it was found that at 9476 m/z, there was a linear relationship between the number of bacterial cells; the relationship between relative intensity (y) and the number of bacterial cells (x) was found to be y = 0.0005 x − 0.00009, and the correlation coefficient was 0.9852.

At the same time, the relative standard deviation values corresponding to the number of different microbial cells were calculated (Table S1). The relative standard deviation values corresponding to the number of different microbial cells were less than 3.29%, which was within the credible range. The results show that the method has good repeatability.

According to a visual inspection of the MALDI-TOF MS profiles (Figure 5A), LB liquid medium seems to have had a more beneficial pre-enrichment effect on the detection of *C. sakazakii* in the simulated environment, and the obtained MALDI-TOF MS spectrum shows a linear relationship between the peak intensities and the number of bacterial cells; this relationship, then, may be useful for practical applications. In previous studies, it has been demonstrated that the different ribosomal protein peaks in MALDI-TOF MS spectra can be used to discriminate pathogenic bacterial strains at the same species level with different molecular typing [35]. The present study, however, provided for the first time an analysis of the linear relationship between the bacterial cell number of *C. sakazakii* and the MALDI-TOF MS peak intensity. This linear relationship suggests the feasibility of the use of MALDI-TOF MS for quantitative as well as qualitative detection of *C. sakazakii*, which is consistent with the quantitative results obtained by Hsieh et al. regarding the quantitative identification of several other classes of pathogenic bacteria [36]. Specifically, the method described here can be applied to the quantitative detection of *C. sakazakii* in environmental samples from PIF production and processing factories.

Figure S1 shows that there are some differences in MALDI-TOF MS spectra between the contaminated PIF samples and the uncontaminated PIF samples after pretreatment. The number of peaks in uncontaminated PIF samples was less than that in contaminated PIF samples, and the spectrum of uncontaminated PIF samples could not be identified, but the spectrum of contaminated PIF samples could be identified as *C. sakazakii.*

The results showed that the accuracy of MALDI-TOF MS identification was still guaranteed by washing only PIF samples contaminated with *C. sakazakii* with PBS and removing the upper layer of fat. Furthermore, other components in PIF do not have a large impact on subsequent MALDI-TOF MS detection. This demonstrated the feasibility of this method for the detection of *C. sakazakii* in PIF samples by MALDI-TOF MS.

The pre-enrichment method was further used to detect *C. sakazakii* in actual PIF samples using MALDI-TOF MS, as shown in Figure 5B. After 4 h of pre-enrichment treatment, the detection limit reached 1.9 × 10^4^ cfu/mL; after 6 h of pre-enrichment treatment, the detection limit was found to reach 2.72 × 10^3^ cfu/mL. At the same time, after analyzing the data of different numbers of bacterial cells under 6 h pre-enrichment, it was found that there was a linear relationship between the number of bacterial cells over the range of 10^3^ to 10^8^ cfu/mL and the intensity of the characteristic MS peak at 9476 *m*/*z*. Here, the relationship was described by the equation y = 0.0009x + 0.0016, and the correlation coefficient was 0.9693.

To further investigate the stability of the method, we calculated the relative standard deviation of the results obtained after multiple tests, and the results are shown in Table S2. When the method was used to detect three different artificially contaminated PIF samples on the market (the detection frequency was five days), the RSD values were all less than 1.78%, indicating that the method had good stability.

Javůrková et al. compared the timeliness of three methods of *Cronobacter* detection (ISO), immunochromatographic test (ICT), and traditional MALDI-TOF MS methods: (1) The traditional ISO method takes 140 h for the detection of *Cronobacter* in PIF samples; (2) ICT methods take 24 h; and (3) the traditional MALDI-TOF MS method takes 46 h [37]. All three methods require pre-enrichment medium (BPW or mLST) to encourage PIF samples. This is more expensive than the pre-enrichment medium (LB) used in this study. However, the novel MALDI-TOF MS method developed in this study only takes 8 h for the detection of *C. sakazakii* in PIF (6 h for pre-enrichment + 2 h for pretreatment and detection). The spectrogram acquisition time for each point was about 0.4 min. The time to process the spectra and analyze the whole sample plate (96 spots) was about 10 min. The detection limit was good.

In addition, the newly described operation is simple and more environmentally friendly than the traditional method. This is because there will be less contamination of *C. sakazakii* that may occur due to the use of inoculation rings to pick single colonies.

## 4. Conclusions

In this study, we developed and optimized a liquid spotting pretreatment method for the MALDI-TOF MS-based detection and identification of *Cronobacter* spp. The optimal parameter conditions were the addition of 25 μL of 70% FA, the application of ultrasound with a power of 350 W and a time of 3 min, and the addition of 75 μL of ACN. Under these conditions, the identification score of *C. sakazakii* reached 1926.42 ± 48.497. This method was associated with consistently strong identification accuracy, and it is applicable to the identification of multiple strains of *C. sakazakii*. Compared with the traditional MALDI-TOF MS method, the detection time of this method can be shortened to 8 h. Meanwhile, the method was evaluated at different frequencies and with different samples, and the overall results showed good stability. Thus, we conclude that this new method is superior to the traditional solid spotting method.

This study also reports the first direct identification and detection of *C. sakazakii* in environmental samples and PIF samples by MALDI-TOF MS. Overall, this method is a useful alternative to traditional MALDI-TOF MS-based pathogen detection and identification methods. Future studies will adapt this method to the detection of mixed samples and samples containing multiple strains of microorganisms.

## Figures and Tables

**Table 1:** Four-factor and three-level response surface methodology design.

Level	70% FA Added Amounts (μL)	Ultrasonic Power (W)	Ultrasonic Time (min)	ACN Added Amounts (μL)
1	10	300	1	40
2	20	350	2	60
3	30	400	3	80

**Table 2:** Box-Behnken design with coded values for the RSM.

No.	70% FA Added Amounts (μL)	Ultrasonic Power (W)	Ultrasonic Time (min)	ACN Added Amounts (μL)	Identification Score
1	10	300	2	60	1770
2	10	350	1	60	1648
3	10	350	2	80	1695
4	10	350	3	60	1464
5	10	350	2	40	1530
6	10	400	2	60	1626
7	20	300	1	60	1721
8	20	300	3	60	1747
9	20	300	2	80	1891
10	20	300	2	40	1534
11	20	350	1	40	1812
12	20	350	1	80	1505
13	20	350	3	40	1553
14	20	350	3	80	1777
15	20	400	1	60	1636
16	20	400	2	40	1696
17	20	400	2	80	1728
18	20	400	3	60	1867
19	30	300	2	60	1601
20	30	350	1	60	1543
21	30	350	2	40	1399
22	30	350	2	80	1877
23	30	350	3	60	1650
24	30	400	2	60	1723
25	20	350	2	60	1879
26	20	350	2	60	1809
27	20	350	2	60	1725
28	20	350	2	60	1901
29	20	350	2	60	1924

**Table 3:** Sum of square, mean square, F-value, R^2^, and lack of fit values of RSM.

Source	Sum of Squares	Degree of Freedom	Mean Square	F Value	R^2^	Significance Level
Model	4.50 × 10^5^	14	32,170.79	3.78	0.0091	Significant
A-70% FA added amounts	300	1	300	0.035	0.8538	
B-ultrasonic power	12	1	12	1.41 × 10^−3^	0.9706	
C-ultrasonic time	3104.08	1	3104.08	0.36	0.5557	
D-ACN added amounts	75,050.08	1	75,050.08	8.81	0.0102	Significant
AB	17,689	1	17,689	2.08	0.1716	
AC	21,170.25	1	21,170.25	2.49	0.1372	
AD	24,492.25	1	24,492.25	2.88	0.1121	
BC	10,506.25	1	10,506.25	1.23	0.2855	
BD	26,406.25	1	26,406.25	3.1	0.1001	
CD	70,490.25	1	70,490.25	8.28	0.0122	Significant
A^2^	1.45 × 10^5^	1	1.45 × 10^5^	17	0.001	Significant
B^2^	3335.06	1	3335.06	0.39	0.5416	
C^2^	64,605.66	1	64,605.66	7.58	0.0155	Significant
D^2^	53,184.66	1	53,184.66	6.24	0.0255	Significant
Residual	1.19 × 10^5^	14	8518.41			
Lack of fit	93,062.58	10	9306.26	1.42	0.3928	Not significant
Pure error	26,195.2	4	6548.8			
Cor total	5.70 × 10^5^	28				

**Table 4:** Identification of *C. sakazakii* by MALDI-TOF MS liquid pretreatment spotting under different medium components.

Culture Medium	Identification Score	Average Value	Standard Deviation
LB-1	2000		
LB-2	1916	1972.000	48.497
LB-3	2000		
NB-1	1943		
NB-2	1988	1938.666	51.636
NB-3	1885		
TSB-1	2000		
TSB-2	1930	1976.666	40.414
TSB-3	2000		

Note: LB 1-3, NB 1-3, and TSB 1-3 in the table are all parallel experiments of the influence of different medium components on the MALDI-TOF MS identification score.

**Table 5:** Comparison of MALDI-TOF MS liquid pretreatment identification results with 16S rDNA identification results.

Strains of Label	Territory	Source	MALDI-TOF MS Identification Result	16S rDNA Identification Result
CE1		Commercially available PIF	*C. sakazakii*	*C. sakazakii*
CE7			*C. sakazakii*	*C. sakazakii*
CE9			*C. sakazakii*	*C. sakazakii*
CE10			*C. sakazakii*	*C. sakazakii*
CE21			*C. sakazakii*	*C. sakazakii*
CE24			*C. sakazakii*	*C. sakazakii*
CE25			*C. sakazakii*	*C. sakazakii*
CE26			*C. sakazakii*	*C. sakazakii*
CE27			*C. sakazakii*	*C. sakazakii*
CE28			*C. sakazakii*	*C. sakazakii*
CE29			*C. sakazakii*	*C. sakazakii*
CE34			*C. sakazakii*	*C. sakazakii*
CE38			*C. sakazakii*	*C. sakazakii*
CE41			*C. sakazakii*	*C. sakazakii*
CE43	Northeastern China		*C. sakazakii*	*C. sakazakii*
CE44			*C. sakazakii*	*C. sakazakii*
CE47			*C. sakazakii*	*C. sakazakii*
CE48			*C. sakazakii*	*C. sakazakii*
CE49			*C. sakazakii*	*C. sakazakii*
CE50			*C. sakazakii*	*C. sakazakii*
CE54			*C. sakazakii*	*C. sakazakii*
CE55			*C. sakazakii*	*C. sakazakii*
CE56			*C. sakazakii*	*C. sakazakii*
CE69			*C. sakazakii*	*C. sakazakii*
CE30		Plant product	*C. sakazakii*	*C. sakazakii*
CE31			*C. sakazakii*	*C. sakazakii*
CE32			*C. sakazakii*	*C. sakazakii*
CE61			*C. sakazakii*	*C. sakazakii*
CE64			*C. sakazakii*	*C. sakazakii*
CE67			*C. sakazakii*	*C. sakazakii*
CE70			*C. sakazakii*	*C. sakazakii*
CE71			*C. sakazakii*	*C. sakazakii*
CE72			*C. sakazakii*	*C. sakazakii*
CE73			*C. sakazakii*	*C. sakazakii*
CE75			*C. sakazakii*	*C. sakazakii*
CE76			*C. sakazakii*	*C. sakazakii*
CE77			*C. sakazakii*	*C. sakazakii*
CE78			*C. sakazakii*	*C. sakazakii*
CE33		Raw material	*C. sakazakii*	*C. sakazakii*
CE58			*C. sakazakii*	*C. sakazakii*
CE59			*C. sakazakii*	*C. sakazakii*
CE36		Fluidized bed powder	*C. sakazakii*	*C. sakazakii*
CE62			*C. sakazakii*	*C. sakazakii*
CE68			*C. sakazakii*	*C. sakazakii*
CE74			*C. sakazakii*	*C. sakazakii*
CE79			*C. sakazakii*	*C. sakazakii*
CE65		Fixed bed	*C. sakazakii*	*C. sakazakii*
CE66			*C. sakazakii*	*C. sakazakii*
CE60		U-shaped valve	*C. sakazakii*	*C. sakazakii*
			*C. sakazakii*	*C. sakazakii*
CE63		Spray dried powder	*C. sakazakii*	*C. sakazakii*
			*C. sakazakii*	*C. sakazakii*
CE51	Northwestern China	Commercially available PIF	*C. sakazakii*	*C. sakazakii*
CE52			*C. sakazakii*	*C. sakazakii*
CE53			*C. sakazakii*	*C. sakazakii*
CE13	Northern China	Commercially available PIF	*C. sakazakii*	*C. sakazakii*
CE14			*C. sakazakii*	*C. sakazakii*
CE15			*C. sakazakii*	*C. sakazakii*
CE16			*C. sakazakii*	*C. sakazakii*
CE17			*C. sakazakii*	*C. sakazakii*
CE18			*C. sakazakii*	*C. sakazakii*
CE19			*C. sakazakii*	*C. sakazakii*
CE20			*C. sakazakii*	*C. sakazakii*
CE22			*C. sakazakii*	*C. sakazakii*
CE23			*C. sakazakii*	*C. sakazakii*
CE11	Eastern China	Commercially available PIF	*C. sakazakii*	*C. sakazakii*
CE12			*C. sakazakii*	*C. sakazakii*
CE8	North China	Commercially available PIF	*C. sakazakii*	*C. sakazakii*
ATCC BAA-894	America	PIF Manufacturing environment	*C. sakazakii*	*C. sakazakii*
ATCC 29004		Commercially available PIF	*C. sakazakii*	*C. sakazakii*
ATCC 29544			*C. sakazakii*	*C. sakazakii*
ATCC 12868			*C. sakazakii*	*C. sakazakii*

## Data Availability

Not applicable.

## Supplementary Materials

The following supporting information can be downloaded at: https://www.mdpi.com/article/10.3390/foods12101981/s1, Figure S1: MALDI-TOF MS spectra of uncontaminated and contaminated PIF samples after pretreatment; Table S1: Relative standard deviations after pretreatment of different artificially contaminated environment sample; Table S2: Relative standard deviations after pretreatment of different artificially contaminated PIF samples in the market.

## References

[1] Liu X., Liu G., Wu Y., Pang X., Wu Y., Niu J., Chen Q., Zhang X. (2021). Transposon sequencing: A powerful tool for the functional genomic study of food-borne pathogens. Trends Food Sci. Technol..

[2] Hong H., Sun C., Wei S., Sun X., Mutukumira A., Wu X. (2020). Development of a real-time recombinase polymerase amplification assay for rapid detection of Salmonella in powdered infant formula. Int. Dairy J..

[3] Yang S., Pei X., Wang G., Yan L., Hu J., Li Y., Li N., Yang D. (2016). Prevalence of food-borne pathogens in ready-to-eat meat products in seven different Chinese regions. Food Control.

[4] Bridier A., Sanchez-Vizuete P., Guilbaud M., Piard J.C., Naitali M., Briandet R. (2015). Biofilm-associated persistence of food-borne pathogens. Food Microbiol..

[5] Chen D., Peng P., Zhou N., Cheng Y., Min M., Ma Y., Mao Q., Chen P., Chen C., Ruan R. (2019). Evaluation of *Cronobacter sakazakii* inactivation and physicochemical property changes of non-fat dry milk powder by cold atmospheric plasma. Food Chem..

[6] Zhang Y., Xie Y., Tang J., Wang S., Wang L., Zhu G., Li X., Liu Y. (2020). Thermal inactivation of *Cronobacter sakazakii* ATCC 29544 in powdered infant formula milk using thermostatic radio frequency. Food Control.

[7] Wang L., Forsythe S.J., Yang X., Fu S., Man C., Jiang Y. (2021). Invited review: Stress resistance of *Cronobacter* spp. affecting control of its growth during food production. J. Dairy Sci..

[8] Badawy B., Gwida M., Sadat A., El-Toukhy M., Sayed-Ahmed M., Alam N., Ahmad S., Ali M.D.S., Elafify M. (2022). Prevalence and Antimicrobial Resistance of Virulent *Listeria monocytogenes* and *Cronobacter sakazakii* in Dairy Cattle, the Environment, and Dried Milk with the In Vitro Application of Natural Alternative Control. Antibiotics.

[9] Luo L., Yi L., Chen J., Liu B., Lü X. (2021). Antibacterial mechanisms of bacteriocin BM1157 against *Escherichia coli* and *Cronobacter sakazakii*. Food Control.

[10] Priego R., Medina L.M., Jordano R. (2011). Bactometer system versus traditional methods for monitoring bacteria populations in salchichon during its ripening process. J. Food Prot..

[11] Xie X., Liu Z. (2021). Simultaneous enumeration of *Cronobacter sakazakii* and *Staphylococcus aureus* in powdered infant foods through duplex TaqMan real-time PCR. Int. Dairy J..

[12] Duan M., Xiao X., Huang Y., Li G., Shan S., Lv X., Zhou H., Peng S., Liu C., Liu D. (2021). Immuno-HCR based on contact quenching and fluorescence resonance energy transfer for sensitive and low background detection of *Escherichia coli* O157:H7. Food Chem..

[13] Amrane S., Lagier J.-C. (2018). Metagenomic and clinical microbiology. Hum. Microbiome J..

[14] Elbir H., Robert C., Nguyen T.T., Gimenez G., El Sanousi S.M., Flock J.-I., Raoult D., Drancourt M. (2013). *Staphylococcus aureus subsp. anaerobius* strain ST1464 genome sequence. Stand. Genom. Sci..

[15] Luo J., Li J., Yang H., Yu J., Wei H. (2017). Accurate Detection of Methicillin-Resistant *Staphylococcus aureus* in Mixtures by Use of Single-Bacterium Duplex Droplet Digital PCR. J. Clin. Microbiol..

[16] Bachli P., Baars S., Simmler A., Zbinden R., Schulthess B. (2022). Impact of MALDI-TOF MS identification on anaerobic species and genus diversity in routine diagnostics. Anaerobe.

[17] Ashfaq M.Y., Da’na D.A., Al-Ghouti M.A. (2022). Application of MALDI-TOF MS for identification of environmental bacteria: A review. J. Environ. Manag..

[18] Oviaño M., Rodríguez-Sánchez B. (2021). MALDI-TOF mass spectrometry in the 21st century clinical microbiology laboratory. Enferm. Infecc. Y Microbiol. Clin..

[19] Moreno E., Miller E., Miller E., Totty H., Deol P. (2018). A novel liquid media mycobacteria extraction method for MALDI-TOF MS identification using VITEK(R) MS. J. Microbiol. Methods.

[20] Sun J., Shi H., Xue Y., Cheng W., Yu M., Ding C., Xu F., Yu S. (2021). Releasing bacteria from functional magnetic beads is beneficial to MALDI-TOF MS based identification. Talanta.

[21] Lau A.F., Walchak R.C., Miller H.B., Slechta E.S., Kamboj K., Riebe K., Robertson A.E., Gilbreath J.J., Mitchell K.F., Wallace M.A. (2019). Multicenter Study Demonstrates Standardization Requirements for Mold Identification by MALDI-TOF MS. Front. Microbiol..

[22] Petry S., Py J.S., Wilhelm A., Duquesne F., Bayon-Auboyer M.H., Morvan H., Gassilloud B. (2019). Evaluation of MALDI-TOF MS and an expanded custom reference spectra database for the identification and differentiation of *Taylorella equigenitalis* and *Taylorella asinigenitalis*. Diagn. Microbiol. Infect. Dis..

[23] Cipolla L., Rocca F., Armitano R.I., Martinez C., Almuzara M., Faccone D., Vay C., Prieto M. (2019). Development and evaluation of an in-house database for quick identification of Burkholderia contaminans by MALDI-TOF MS. Rev. Argent Microbiol..

[24] Girard V., Mailler S., Welker M., Arsac M., Celliere B., Cotte-Pattat P.J., Chatellier S., Durand G., Beni A.M., Schrenzel J. (2016). Identification of *mycobacterium* spp. and *nocardia* spp. from solid and liquid cultures by matrix-assisted laser desorption ionization-time of flight mass spectrometry (MALDI-TOF MS). Diagn. Microbiol. Infect. Dis..

[25] Oviano M., Rodriguez-Sanchez B., Gomara M., Alcala L., Zvezdanova E., Ruiz A., Velasco D., Gude M.J., Bouza E., Bou G. (2018). Direct identification of clinical pathogens from liquid culture media by MALDI-TOF MS analysis. Clin. Microbiol. Infect..

[26] Stevenson L.G., Drake S.K., Murray P.R. (2010). Rapid identification of bacteria in positive blood culture broths by matrix-assisted laser desorption ionization-time of flight mass spectrometry. J. Clin. Microbiol..

[27] Liu J., Liu Y., Gao M., Zhang X. (2012). High throughput detection of tetracycline residues in milk using graphene or graphene oxide as MALDI-TOF MS matrix. J. Am. Soc. Mass. Spectrom..

[28] Cunsolo V., Muccilli V., Saletti R., Foti S. (2013). MALDI-TOF mass spectrometry for the monitoring of she-donkey’s milk contamination or adulteration. J. Mass. Spectrom..

[29] Aseev L.V., Boni I.V. (2011). Extraribosomal functions of bacterial ribosomal proteins. Mol. Biol..

[30] Guo Z., Zhao B., Li H., Miao S., Zheng B. (2019). Optimization of ultrasound-microwave synergistic extraction of prebiotic oligosaccharides from sweet potatoes (*Ipomoea batatas* L.). Innov. Food Sci. Emerg. Technol..

[31] Dranca F., Oroian M. (2016). Optimization of ultrasound-assisted extraction of total monomeric anthocyanin (TMA) and total phenolic content (TPC) from eggplant (*Solanum melongena* L.) peel. Ultrason. Sonochem..

[32] Raethong N., Wang H., Nielsen J., Vongsangnak W. (2020). Optimizing cultivation of Cordyceps militaris for fast growth and cordycepin overproduction using rational design of synthetic media. Comput. Struct. Biotechnol. J..

[33] Deepika D., Saigal K., Ghosh A., Saikia D., Balaji V., John J., Kang G. (2020). Spatial cluster analysis of invasive typhoidal Salmonella infections from paediatric population in North India. Int. J. Infect. Dis..

[34] Wang Q., Zhao X.J., Wang Z.W., Liu L., Wei Y.X., Han X., Zeng J., Liao W.J. (2017). Identification of Cronobacter species by matrix-assisted laser desorption/ionization time-of-flight mass spectrometry with an optimized analysis method. J. Microbiol. Methods.

[35] Dubois D., Leyssene D., Chacornac J.P., Kostrzewa M., Schmit P.O., Talon R., Bonnet R., Delmas J. (2010). Identification of a variety of Staphylococcus species by matrix-assisted laser desorption ionization-time of flight mass spectrometry. J. Clin. Microbiol..

[36] Hsieh S.Y., Tseng C.L., Lee Y.S., Kuo A.J., Sun C.F., Lin Y.H., Chen J.K. (2008). Highly efficient classification and identification of human pathogenic bacteria by MALDI-TOF MS. Mol. Cell Proteom..

[37] Javůrková B., Blažková M., Fukal L., Rauch P. (2012). Rapid detection of genus Cronobacter in powdered infant formula milk. Eur. Food Res. Technol..

