# Supplementary material for: A Method to Directly Identify Cronobacter sakazakii in Liquid Medium by MALDI-TOF MS
Source: Foods. 2023 May 12;12(10):1981. doi: 10.3390/foods12101981 (PMC10217744; doi:10.3390/foods12101981)
Supplement: Supplementary file 1 [file foods-12-01981-s001.zip › foods-2235898-supplementary/Figure S1.docx]

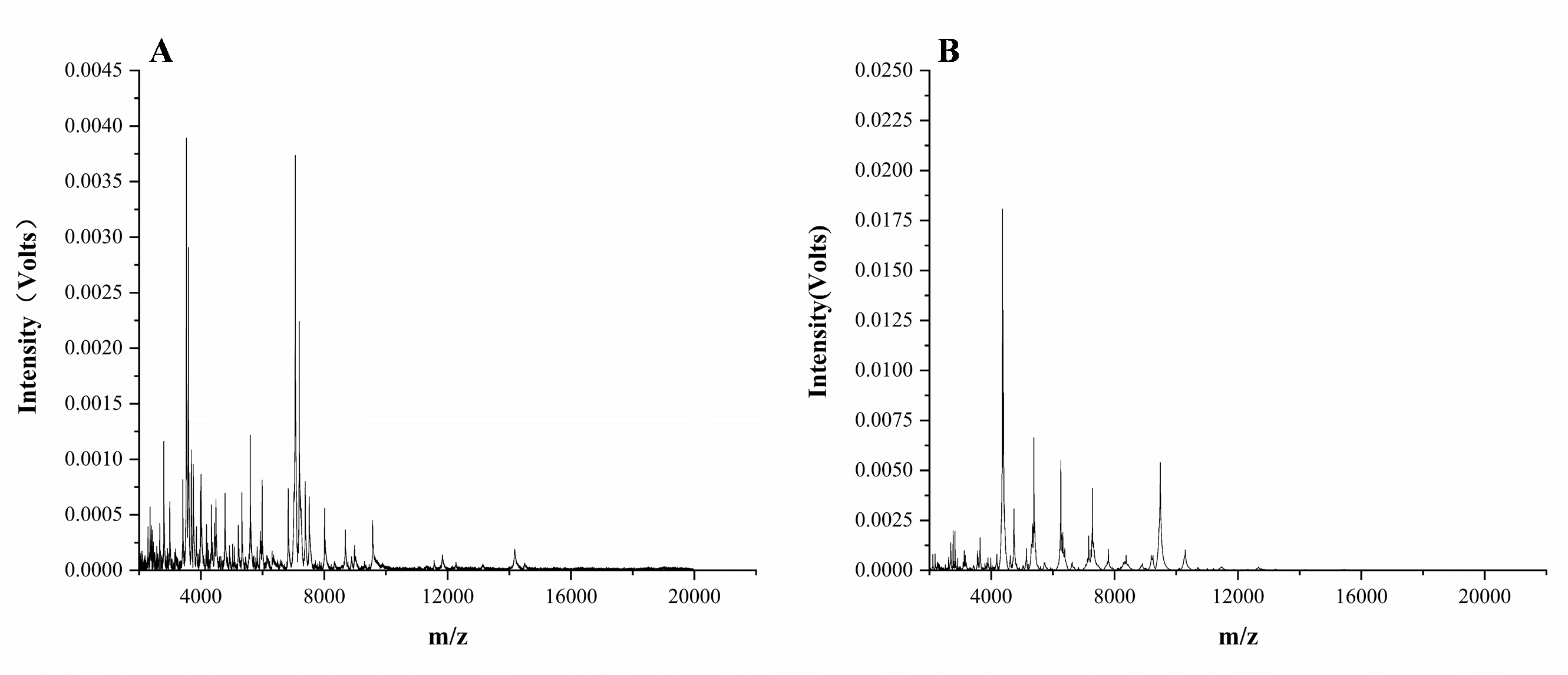


**Figure S1.** MALDI-TOF MS spectra of uncontaminated and contaminated PIF samples after pretreatment

(**A**) MALDI-TOF MS spectra of contaminated PIF samples. (**B**) MALDI-TOF MS spectra of uncontaminated blank PIF samples after pretreatment.
