# Supplementary material for: A Method to Directly Identify Cronobacter sakazakii in Liquid Medium by MALDI-TOF MS
Source: Foods. 2023 May 12;12(10):1981. doi: 10.3390/foods12101981 (PMC10217744; doi:10.3390/foods12101981)
Supplement: Supplementary file 1 [file foods-12-01981-s001.zip › foods-2235898-supplementary/Table S1.docx]

**Table S1.** Relative standard deviations after pretreatment of different artificially contaminated environment sample

| **cfu/mL** | **Intensity (Volts) of 9476 m/z** | | | **average value** | **Standard deviation** | **RSD (%)** |
| --- | --- | --- | --- | --- | --- | --- |
|  | **Time 1** | **Time 2** | **Time 3** |  |  |  |
| 10^8^ | 0.00386 | 0.00387 | 0.00384 | 0.00386 | 1.60416E-05 | 0.41 |
| 10^7^ | 0.00311 | 0.00311 | 0.00328 | 0.00317 | 9.81495E-05 | 3.09 |
| 10^6^ | 0.00245 | 0.00254 | 0.00254 | 0.00251 | 5.19615E-05 | 2.07 |
| 10^5^ | 0.00224 | 0.00223 | 0.00223 | 0.00223 | 5.7735E-06 | 0.25 |
| 10^4^ | 0.00186 | 0.00189 | 0.00186 | 0.00187 | 1.73205E-05 | 0.92 |
| 10^3^ | 0.00128 | 0.00131 | 0.00128 | 0.00129 | 1.73205E-05 | 1.34 |
| 10^2^ | 0.00103 | 0.00103 | 0.00109 | 0.00105 | 3.4641E-05 | 3.29 |
| 10^1^ | 0.00029 | 0.00285 | 0.00029 | 0.00029 | 5.50757E-06 | 1.89 |
