# Supplementary material for: A Method to Directly Identify Cronobacter sakazakii in Liquid Medium by MALDI-TOF MS
Source: Foods. 2023 May 12;12(10):1981. doi: 10.3390/foods12101981 (PMC10217744; doi:10.3390/foods12101981)
Supplement: Supplementary file 1 [file foods-12-01981-s001.zip › foods-2235898-supplementary/Table S2.docx]

**Table S2.** Relative standard deviations after pretreatment of different artificially contaminated PIF samples in the market

| **Sample** | **cfu/mL** | **Intensity (Volts) of 9476 m/z** | | | | | **average value** | **Standard deviation** | **RSD (%)** |
| --- | --- | --- | --- | --- | --- | --- | --- | --- | --- |
|  |  | **Day 1** | **Day 2** | **Day 3** | **Day 4** | **Day 5** |  |  |  |
| Sample A | 10^8^ | 0.00561 | 0.00557 | 0.00563 | 0.00561 | 0.00559 | 0.00933 | 2.28×10^-5^ | 0.24 |
|  | 10^7^ | 0.00520 | 0.00517 | 0.00518 | 0.00517 | 0.00519 | 0.00863 | 1.30×10^-5^ | 0.15 |
|  | 10^6^ | 0.00441 | 0.00449 | 0.00447 | 0.00450 | 0.00443 | 0.00743 | 3.87×10^-5^ | 0.52 |
|  | 10^5^ | 0.00297 | 0.00289 | 0.00294 | 0.00292 | 0.00295 | 0.00489 | 3.05×10^-5^ | 0.62 |
|  | 10^4^ | 0.00258 | 0.00252 | 0.00261 | 0.00259 | 0.00260 | 0.00430 | 3.54×10^-5^ | 0.82 |
|  | 10^3^ | 0.00094 | 0.00095 | 0.00094 | 0.00095 | 0.000945 | 0.00158 | 3.16×10^-6^ | 0.19 |
| Sample B | 10^8^ | 0.00558 | 0.00563 | 0.00556 | 0.00561 | 0.00559 | 0.00932 | 2.70×10^-5^ | 0.28 |
|  | 10^7^ | 0.00519 | 0.00518 | 0.00515 | 0.00517 | 0.00521 | 0.00863 | 2.24×10^-5^ | 0.26 |
|  | 10^6^ | 0.00438 | 0.00446 | 0.00440 | 0.00441 | 0.00445 | 0.00736 | 3.39×10^-5^ | 0.46 |
|  | 10^5^ | 0.00301 | 0.00295 | 0.00299 | 0.00297 | 0.00300 | 0.00497 | 2.41×10^-5^ | 0.48 |
|  | 10^4^ | 0.00259 | 0.00257 | 0.00258 | 0.00261 | 0.00257 | 0.00430 | 1.67×10^-5^ | 0.39 |
|  | 10^3^ | 0.00095 | 0.00094 | 0.00093 | 0.00094 | 0.000943 | 0.00157 | 8.35×10^-6^ | 0.53 |
| Sample C | 10^8^ | 0.00569 | 0.00572 | 0.00566 | 0.00568 | 0.00566 | 0.00947 | 2.49×10^-5^ | 0.26 |
|  | 10^7^ | 0.00518 | 0.00520 | 0.00522 | 0.00519 | 0.00523 | 0.00867 | 2.07×10^-5^ | 0.24 |
|  | 10^6^ | 0.00434 | 0.00442 | 0.00445 | 0.00447 | 0.00439 | 0.00735 | 5.13×10^-5^ | 0.70 |
|  | 10^5^ | 0.00299 | 0.00295 | 0.00297 | 0.00298 | 0.00295 | 0.00494 | 1.79×10^-5^ | 0.36 |
|  | 10^4^ | 0.00249 | 0.00252 | 0.00256 | 0.00253 | 0.00250 | 0.00420 | 2.74×10^-5^ | 0.65 |
|  | 10^3^ | 0.00094 | 0.00094 | 0.00094 | 0.00094 | 0.00101 | 0.00159 | 2.86×10^-5^ | 1.78 |
